# Quality of Chatbot Responses to the Most Popular Questions Regarding Erectile Dysfunction

**DOI:** 10.5152/tud.2025.24098

**Published:** 2025-01-03

**Authors:** İrfan Şafak Barlas, Lütfi Tunç

**Affiliations:** Clinic of Urology, Ankara Acibadem Hospital, Ankara, Türkiye

**Keywords:** Artificial intelligence, Chatbot, Global Quality Score, ChatGPT, Gemini, Erectile dysfunction

## Abstract

**Objective:**

Erectile dysfunction (ED) is a common cause of male sexual dysfunction. We aimed to evaluate the quality of ChatGPT and Gemini’s responses to the most frequently asked questions about ED.

**Methods:**

This study was conducted as a cross-sectional, observational study. Google Trends was used to determine the most frequently asked questions on the internet. ChatGPT-3.5 and Gemini were compared for these chatbots’ answers to the questions about ED. Two urologists with board certificates assessed the quality of responses using the Global Quality Score (GQS).

**Results:**

Fifteen questions about ED were included according to the Google Trends. ChatGPT was able to answer all the questions systematically, whereas Gemini could not answer two questions. Upon assessing the quality of the responses provided by both researchers with the GQS, it was observed that the frequency of low-quality responses from Gemini exceeded that of ChatGPT. The agreement between researchers was 92% for ChatGPT and 95% for Gemini.

**Conclusion:**

Despite the expeditious and comprehensive answers provided by chatbots, we identified inadequacies in their responses related to ED. In their current state, they cannot replace the patient-centered approach of healthcare professionals and require further development.

**Main Points**

- It has been observed that both ChatGPT and Gemini may provide misleading answers to patients.
- ChatGPT answered all of the questions, but there were a few that Gemini could not answer.
- ChatGPT had a higher number of excellent and good-quality responses than Gemini.
- The number of poor and generally poor quality responses from Gemini was greater than that from ChatGPT.
- At the end of their responses, both Gemini and ChatGPT emphasize the importance of consulting a healthcare professional.

## Introduction

Erectile dysfunction (ED) is a medical condition characterized by the inability to achieve or maintain a penile erection that is adequate for the purpose of having successful vaginal intercourse.^1^ In 2000, it was estimated that ED affected up to 30 million men in the United States and 150 million men worldwide, and its prevalence is increasing rapidly. By 2025, it is anticipated that 322 million men will be affected worldwide.^2,3^

Erectile function is dependent on a complex cooperation between vascular and neural mechanisms. The main source of blood supply to the penis is the internal pudendal artery, which delivers blood through the cavernosal branches. The occurrence of arousal prompts the activation of parasympathetic activity coming from the sacral segments of the spinal cord. This activation sets in motion a series of events aimed at the release of nitric oxide and the subsequent elevation of intracellular cyclic guanosine monophosphate levels, inducing vascular smooth muscle relaxation and leading to enhanced blood flow into the corpora cavernosa. As a consequence, the pressure within the cavernosal region increases, leading to the occurrence of an erection. Consequently, erectile dysfunction can arise due to any mechanism that hinders the neural or vascular pathways involved in the erection process.^4^ Therefore, several factors that impact the vascular and nervous systems, especially diabetes mellitus, cardiovascular diseases, and psychogenic disorders, have been found to be strongly linked with the occurrence of erectile dysfunction.^5-7^

The utilization of artificial intelligence (AI) in large language models (LLMs) enables students and researchers, as well as patients, to conveniently access information.^8^ The Chat Generative Pre-trained Transformer (ChatGPT) has been developed by OpenAI with the purpose of creating text that resembles human language. This resource in question has garnered public interest and is readily available. Nevertheless, since the database used in training ChatGPT (San Francisco, Calif., USA) is not publicly available, there are concerns about the accuracy and quality of the information obtained.^9^ An additional significant conversational agent powered by AI is Gemini (Mountain View, Calif., USA), which was created by Google. The purpose of this study was to evaluate the quality of ChatGPT and Gemini’s responses to the most frequently asked questions about ED, one of the most popular topics in the field of urology, that were found on the Internet.

## Material and Methods

This study was conducted as a cross-sectional, observational study. Since there were no human or animal subjects, ChatGPT-3.5 and Gemini are free to everyone, ethical approval and informed consent of patients were not required.

### Selection of Questions

Google Trends was used to determine the most frequently asked questions on the internet. The term “erectile dysfunction” was searched on Google Trends in September 2023. The filters were selected as “Worldwide,” “Last 5 years”, “Health,” and “Google Web Search” and listed as most viewed. Duplicate questions, meaningless words, single words without a question pattern, questions unrelated to the topic, and questions in a language other than English were excluded.

### Evaluation of the Answers

Each of these questions was posed to ChatGPT-3.5 and Gemini in English as separate, independent prompts using the “New Chat” function provided in September 2023, and the responses were recorded. They were evaluated by two board-certified researchers, each of whom had at least 10 years of experience in the field of urology. The European Association of Urology (EAU) and the American Urological Association (AUA) guidelines were used as references in evaluating the answers.^10,11^ In addition, the Global Quality Score (GQS) was used for the evaluation of the quality of answers. GQS is a 5-point Likert scale based on the quality of information, the flow, and ease of using information, which was first used by Bernard et al. According to the GQS: 1—poor, 2—generally poor, 3moderate, 4—good, and 5—excellent quality.^12^ A reliability analysis was conducted to determine the agreement between researchers.

### Statistical Analysis

Statistical Package for the Social Sciences (SPSS), version 22.0 (IBM SPSS Corp.; Armonk, NY, USA), was used for statistical analysis. Intra-class correlation was performed to determine inter-rater reliability. Descriptive data were presented as numbers and percentages.

## Results

After applying the exclusion criteria, fifteen questions about ED were included in our study. ChatGPT was able to answer all the questions, whereas Gemini could not answer two questions.

The questions and some strengths and limitations of the responses given by ChatGPT (Table 1) and Gemini (Table 2) were summarized. The evaluation of the answers given by ChatGPT and Gemini according to GQS was presented in Table 3. The agreement between researchers in evaluating the answers to the questions was 92% (ICC: 0.919, *P* < .001) for ChatGPT and 95% (ICC: 0.950, *P* < .001) for Gemini.

## Discussion

In this study, two participants reviewed ChatGPT’s and Geminin’s responses to frequently asked questions about ED. A comparative analysis of ChatGPT and Gemini based on their responses to questions regarding ED revealed that each had strengths and limitations in comparison to the other. Both participants consistently provided similar answers without expressing opposing views. When the participants assigned different scores, the ratings remained relatively close, with the majority assigning the same scores. As indicated in the existing literature, our study also found that they used an established algorithm to provide easy-to-understand responses to the questions.^13,14^ Additionally, both ChatGPT and Gemini highlight the importance of consulting a healthcare professional at the end of their responses.

Another observation from our study indicated that although the chatbots provided sufficient responses to general descriptive questions, they gave insufficient and even misleading responses regarding ED treatment and prevention, as well as the factors that cause ED. While chatbots can be useful in assisting, guiding patient care, and describing the ability to answer patient-centered questions, their accuracy in doing so must be examined and evaluated.^15^ Because chatbots obtain their responses from databases using an advanced reinforcement learning system, these sources are primarily medical texts, research papers, guidelines from health organizations, and other official medical-related resources. They may also utilize old data sources that no longer contain accurate medical information or have been altered.^16-18^

The chatbots do not personalize or modify their responses based on the person asking the question; rather, they provide similar responses to similar questions based on predefined patterns. Howard et al^19^ also concluded that a lack of situational awareness is one of the main challenges to the effective use of ChatGPT in clinical practice. When the data provided is “insufficient” or “incompatible,” a quality check or confidence level filter may be useful for directing patients to a healthcare professional. The chatbots are an automation system and are useful in decreasing physician workload under physician guidance and control, by preventing human-induced incomplete information in patient information and producing standard answers; they can greatly facilitate direct patient-physician interaction and thus improve the quality of care. Additionally, when Jackson et al^20^ conducted a bibliometric analysis of the information they examined regarding prostate cancer, it was noted that these resources exhibit characteristics such as being outdated, biased, challenging for patients to comprehend, or lacking essential information.

Chatbots cannot discern specific rules, cultural differences, or terms of support that may be relevant to a particular region or country unless the question is directly phrased as being region-specific.^8^ When a patient’s country or region follows different recommendations, it can be confusing for the patient. In this instance, to obtain accurate and regionally relevant information, it is essential to confirm it with qualified healthcare professionals or official medical sources containing reliable procedures. Similarly, our research supports the idea that many of the treatment options recommended by chatbots for ED are inaccessible in particular areas. More research is required to improve the accuracy of medical information generated by AI across a range of question types and with a variety of healthcare professional groups.

In studies evaluating the intention of chatbots’ response skills, the selection of questions is usually subjective, and some studies incorporate inquiries posed by specialists in the respective field as opposed to patients.^21-25^ However, to determine the research questions, we focused on the most frequently searched queries using Google Trends. Unlike previous studies, the use of the Google Trends tool allowed for an objective assessment in identifying the most commonly asked questions. But it is important to note that trending questions on Google Trends may vary depending on the period, language, and region.^26^ Moreover, the replacement of Bard and the release of Gemini in place of Bard by Google implied not only a modification in title but also a revision in substance. Certain studies conducted prior to the release of Google Gemini inaccurately ascribed the results retrieved from Bard queries to Gemini, claiming that Gemini was recently renamed Bard. Also, they assessed Bard’s responses as Gemini’s responses.^27,28^ Evaluating Gemini according to the responses supplied by Bard might be inappropriate. As a result, in our research, we assessed the responses presented by Gemini subsequent to the debut of Google Gemini.

Gemini did not provide answers to some of the questions we asked in our study, indicating that it could not respond to them. In contrast to ChatGPT, Gemini provided responses to questions regarding certain subjects where insufficient scientific evidence existed by accurately emphasizing the scarcity of medical proof. Furthermore, when the two researchers assessed the quality of ChatGPT’s and Gemini’s answers to ED questions, both of them found that the excellent and good quality answers of ChatGPT were higher than Gemini’s excellent and good quality answers. Due to the recent release of Gemini, one of very rare studies comparing Gemini and ChatGPT’s responses to cardiology-related questions revealed both chatbots to perform highly successfully, with no significant superiority observed between them.^24^ Before Google Gemini was introduced, studies comparing it to Google Bard and ChatGPT concluded that ChatGPT had much more accurate responses and was easier to understand than Bard.^21-23^ However, there were studies arguing that ChatGPT had less specificity and accuracy than Bard.^29,30^ This suggests that the success rate of chatbots may vary depending on the subject matter. It is known that chatbots improve themselves on a topic as they are asked questions, providing more accurate and current responses. Another hypothesis is that because ChatGPT has been in use for a longer period than Gemini, it might be able to give higher-quality answers.

Through regular updates and new versions, chatbots remain equipped with the most up-to-date information. They also offer patients quick and accessible support when making health decisions. By providing accurate advice, chatbots can potentially guide patients until they reach medical care. Relying solely on chatbot advice can lead to self-misdiagnosis, treatment delays, and reduced interaction with healthcare providers, ultimately causing further delays in medical care if the chatbot provides incorrect guidance. Furthermore, privacy concerns arise when sharing personal data, and biased training data could result in misleading information. In cases related to ED, chatbots may serve as a more approachable resource for patients hesitant to consult professionals, though incorrect treatment guidance could lead to serious morbidity.^9^

Our study has some limitations. Initially, we identified the context of questions that met the criteria from the most frequently asked queries on Google Trends, focusing on a specific set of questions. We included the most frequently asked 15 questions in the analysis because we observed that the content of the questions beyond the first 15 was highly similar and repetitive. While our sample consisted of 15 questions, such a limited sample may not fully represent the broader range of inquiries users might have about ED. Additionally, it would be premature to assess the inadequacy of chatbots based on this limited question set. Furthermore, we asked our questions at a particular time and based the responses on that time. However, as AI programs are updated and developed, the responses may change over time. Also, in our study, we examined ChatGPT3-5 and Gemini, although there are many chatbots that are increasing day by day. Our study did not include an evaluation of the paid chatbot version, ChatGPT4-0, because there was a widely used, freely accessible alternative available that did not necessitate payment.

### Future Directions

Future research could expand by evaluating a broader set of questions, incorporating more AI models, and involving additional participants, including healthcare professionals and patients. A long-term study should also be planned to assess how chatbot responses evolve over time with new model versions and updates. Additionally, such studies could evaluate chatbot performance across a wider range of ED-related topics, considering regional and societal differences for a more comprehensive evaluation.

Due to the vast number of different chatbots available, our study focused on two of the most widely accessible and commonly used models. We conducted this analysis by presenting the opinions of two healthcare professionals specializing in ED. Future steps could involve the development of chatbots guided and supervised by a diverse, multilingual, and multicultural team of healthcare professionals. These chatbots should use reliable medical content databases, incorporate evidence-based, current guidelines, and integrate real-time data updates or enhanced contextual understanding, particularly when dealing with conflicting information. Such advancements may enhance AI’s contribution to healthcare by improving response accuracy, timeliness, and reliability.

Despite the expeditious and comprehensive responses provided, inadequacies were detected in the answers given by chatbots related to ED, which is popular health-related topic. Furthermore, Gemini’s responses of poor quality were found to be higher than those of ChatGPT. Our findings indicate that they cannot substitute the patient-centered approach of healthcare professionals with their current format; therefore, further development is required.

## Figures and Tables

**Table 1.** The Questions and Strengths and Limitations of ChatGPT’s Responses

Questions	Strengths	Limitations
What is erectile dysfunction?	It was thoroughly explained and consistent with the literature.	
What are the causes of erectile dysfunction?		Among the drugs that cause ED, ChatGPT also added medications prescribed for prostate conditions, but there is no evidence that prostate medications cause erectile dysfunction. Antipsychotics, antiandrogens, narcotics, and anabolic steroids are not mentioned despite their significant role in the etiology of erectile dysfunction.
Does porn cause erectile dysfunction?		Although ChatGPT speculated that potential psychological, desensitization, neurological, and relationship issues could contribute to ED, there is no evidence to support the claim that watching pornographic materials causes ED.
How to cure erectile dysfunction?	The treatment and recommendations were appropriately addressed, with no misleading information.	Shockwave therapy, platelet-rich plasma, botulinum neurotoxin A, and penile revascularization surgeries, which are in the guidelines, are not mentioned.
What are pills for erectile dysfunction?	Similar to guidelines, PDE5I were recommended as oral drugs for the treatment of ED, and unproven treatments were not mentioned.	In particular, information should be given that serious side effects may occur in the uncontrolled use of drugs and uncontrolled use should be discouraged.
Can high blood pressure cause erectile dysfunction?	It was well-explained and consistent with the literature.	
Can food cause erectile dysfunction?	Without deceiving, ChatGPT provided nutritional recommendations that were both extremely accurate and detailed.	
Are there any foods that can be good for erectile dysfunction?		Citrus fruits, berries, nuts, seeds, whole grains, dark chocolate, pomegranates, garlic, watermelon, and green tea were all recommended by ChatGPT. The recommendations have no clinical significance. There is a lack of established guidelines indicating that the use of some of the nutritional products specifically mentioned here is beneficial, and there are no significant studies proving their effectiveness.
Is there any relationships between COVID and erectil dysfunction?		Although ChatGPT states that COVID-19 can induce ED, no evidence suggests that COVID-19 patients who do not develop cardiac side effects experience ED. Given the stronger correlation between cardiac effects and COVID-19, it has been hypothesized that conditions that induce cardiac side effects may also cause ED. Hence, it is more precise to assert that erectile dysfunction might ensue subsequent to cardiac complications induced by COVID-19.
Is diabetes caused by Erectile dysfunction?	The relationship and pathophysiology of DM and ED were explained accurately.	
What is tadalafil?		Tadalafil is safe to use, according to ChatGPT; however, it should be avoided when taken together with organic nitrates or NO donors, as well as antidepressants, antifungals, antihypertensives, and HIV/AIDS medications, as they may cause severe side effects. It is also stated that the duration of action for tadalafil is 36 hours; however, for tadalafil 20 mg, the duration of action is 36 hours and 24 hours for tadalafil 5 mg tablets. Additionally, it has been mentioned that it may be utilized to treat BPH; however, it is important to mention that for daily use, only the 5 mg dose is appropriate for BPH treatment.
Are there any special exercise types for erectile dysfunction?		ChatGPT suggested that pelvic floor exercises, in addition to many physical exercises, would be beneficial for ED. The benefit of pelvic floor exercise recommendations that are not included in the guidelines and have no cause-and-effect relationship is very limited.
Are there any vitamins for erectile dysfunction?	Possible mechanisms of action of vitamins that may reduce ED have been well explained.	Supplements, including zinc, folic acid, and vitamins B3, C, D, and E, according to ChatGPT, would be advantageous in the treatment of erectile dysfunction. However, modifying dietary habits and engaging in regular physical activity are adequate; supplementation is only required in instances of deficiency; otherwise, the efficacy of vitamin supplements is not significant. Although there are findings suggesting that ginseng alone may be beneficial, this wasn’t stated. Additionally, while there are findings suggesting that L-arginine may be beneficial when used together with PDE5Is, this wasn’t mentioned either.
What are the tests for erectile dysfunction diagnosis?		ChatGPT suggested that, in addition to a penis and testicle examination, a blood pressure measurement be included in the physical examination he advised for ED diagnosis. Furthermore, it ought to have addressed the prostate exam and weight assessment. Although it was stated that Doppler ultrasound should be performed on the patients’ penises, it is advised that Penile Duplex Ultrasound imaging be used following the injection instead of standard Doppler ultrasound imagining.
Are there any relationships between premature ejaculation and erectile dysfunction?	The pathophysiology of the relationship between ED and PE was well explained.	According to ChatGPT, the coexistence of ED and PE is common due to overlapping risk factors, including hypertension, but there is no evidence that hypertension causes PE. Furthermore, it was stated that PE and ED often occur together. One similarity pointed out by ChatGPT was the usage of SSRIs in the treatment of both disorders. However, while SSRIs are not utilized in the treatment of ED, they are among the causes of ED.

PE, premature ejaculation; AIDS, acquired immune deficiency syndrome; BPH, benign prostatic hyperplasia; ED, erectile dysfunction; ChatGPT, Chat Generative Pre-trained Transformer; COVID-19, Coronavirus Disease 2019; DM, diabetes mellitus; HIV, human immunodeficiency virüs; NO, nitric oxide; PDE5I, phosphodiesterase type 5 Inhibitor; SSRI, selective serotonin reuptake inhibitor.

**Table 2.** The Questions and Strengths and Limitations of Gemini’s Responses

Questions	Strengths	Limitations
What is erectile dysfunction	Answered in a simple and clear manner.	Indicated that erectile dysfunction is present in more than half of sexual relationships, but this is controversial as there is widely varying data on this subject.
What are the causes of erectile dysfunction?	Disclosed the sources referred to, presenting certain information obtained from randomized controlled trials accessed through reliable search engines with access to the Medline database, and approciately cited them.	While discussing information obtained from promotional websites, antihistamines were mentioned, although they are not considered a primary factor in the etiology of ED. Additionally, anabolic steroids, antipsychotics, antiandrogens, and significant factors such as smoking and alcohol use were not mentioned.
Does porn cause erectile dysfunction?	Provided a comprehensive and well-explained response, giving accurate information. He acknowledged the possibility of speculations but emphasized the lack of scientific evidence to support them.	
How to cure erectile dysfunction?	Responded by stating that ED is a complex condition with various contributing factors, and emphasized the importance of seeking professional medical help for proper diagnosis and treatment. It also advised against pursuing treatments outside of doctor supervision for individuals with ED complaints, accurately conveying lifestyle changes that the patient can undertake on their own.	
What are pills for erectile dysfunction?		It answered as “*I am unable to recommend or discuss specific medications for erectile dysfunction. Erectile dysfunction medication should only be taken under the supervision of a licensed physician, as they can interact with other medications you are taking and have serious side effects.*”
Can high blood pressure cause erectile dysfunction?	Provided sufficient information that directed the patient appropriately.	
Can food cause erectile dysfunction?	Gave recommendations in a simple and easily understandable manner, avoiding complexity.	Stated that red meat, high in saturated fat, can contribute to erectile dysfunction. However, the advice to avoid meat consumption may be subject to misinterpretation. The information that avoiding red meat is necessary to prevent ED is not accurate.
Are there any foods that can be good for erectile dysfunction?	Stated that there isn’t a specific food that miraculously cures ED, but it was emphasized that certain foods can contribute to a heart-healthy diet, which is also beneficial for erectile dysfunction.	Suggested uniform dietary recommendations include consuming watermelon, spinach, fibrous foods, fatty fish, berries, fruits, vegetables, whole grains, and particularly healthy fats, nuts, and seeds. This information has the potential for misinterpretation, and these recommendations may pose risks when not tailored to individual characteristics. In uncontrolled use, their effectiveness and reliability are questionable.
Is there any relationships between covid and erectil dysfonction?	The potential consequences of post-COVID erectile dysfunction were accurately categorized as being caused by inflammation, vascular disorders, and psychological factors.	Stated that COVID-19 could lead to erectile dysfunction. However, there is no strong evidence that ED develops in COVID-19 patients who do not have cardiovascular side effects. It is more accurate to say that individuals who develop cardiovascular complications may also lead to ED.
Is diabetes caused to erectile dysfunction?		Although one of the most common complications of diabetes is ED, Gemini mentioned that diabetes itself does not cause ED. Instead, it may be more frequent in those with diabetes due to vascular and nerve damage. The information was derived from untrustworthy online sources with dubious scientific content and sources.
What is tadalafil?		It didn’t respond and said,”*I*’*m just a language model, so I can*’*t help you with that.*”
Are there any special exercise types for erectile dysfunction?	Suggested that aerobic exercise, strength training, yoga, and Pilates would be beneficial for ED. Sedentary lifestyles increase the risk of ED, while physical activities are known to provide benefits, so these suggestions could be helpful.	Alongside exercise suggestions that could be beneficial, Gemini also suggested Kegel exercises as the first recommendation. However, it is important to note that there is no evidence to support the benefits of Kegel exercises for ED. Gemini advised patients to “*tighten the muscles you would use to stop urination midstream for 3 seconds,*” but this has no proven benefit for ED.
Are there any vitamins for erectile dysfunction?		Recommended that would be beneficial for ED include folate, vitamin C, and vitamin D when there is a deficiency. It is known that replacing deficient vitamins can be beneficial for ED; however, there is no benefit to supplementation in the absence of deficiencies. Additionally, there is no evidence to suggest that folate, vitamin C, and vitamin D are superior to other vitamins in the treatment of ED. Furthermore, while the benefits of ginseng and L-arginine have been demonstrated in individuals with ED, they have not been mentioned.
What are the tests for erectile dysfunction diagnosis?		Proposed conducting a urine test, which is not relevant for diagnosing ED and has a negligible effect on the diagnosis. Moreover, the guidelines propose that intravenous injection tests offer diagnostic advantages; however, Gemini neglects discussing this aspect. It is suggested to perform Doppler Ultrasound Imaging, emphasized as a painless and simple test; however, guidelines recommend not a standard Doppler Ultrasound but rather a Penile Duplex Ultrasound (DUS) after injection.
Are there any relationships between prematüre ejaculation and erectile dysfunction?	Emphasized the frequent co-occurrence of ED and PE.	Provided inaccurate information while expounding on the frequent coexistence of ED and PE. Furthermore, it cited the fact that the same medications pose risks for both conditions as an example of their frequent co-occurrence. However, while SSRIs may cause ED, there is no information suggesting they cause PE; in fact, they often delay ejaculation. Additionally, another example it provided to strengthen the association between ED and PE mentioned that heart diseases could be contributory to both conditions. While heart diseases may be a predisposing factor for ED, they are not for PE.

COVID-19, Coronavirus Disease 2019; DM, diabetes mellitus; EAU, European Association of Urology; ED, erectile dysfunction; PDE5I, phosphodiesterase type 5 inhibitor; PE, premature ejaculation.

**Table 3.** The Evaluation of ChatGPT’s and Gemini’s Responses According to the GQS

Score	Global Score Description	ChatGPT’s Responses According to Researcher 1	ChatGPT’s Responses According to Researcher 2	Gemini’s Responses According to Researcher 1	Gemini’s Responses According to Researcher 2
1	Poor quality, poor flow of the site, most information missing, not at all useful for patients	0	0	13.3%	13.3%
2	Generally poor quality, poor flow, some information listed, but many important topics missing of very limited use to patients	6.7%	0	13.3%	0
3	Moderate quality, suboptimal flow, some important information is adequately discussed, but others poorly discussed, somewhat useful to patients	26.7%	33.3%	33.3%	40.0%
4	Good quality and generally good flow, most of the relevant information is listed, but some topics are not covered, useful to patients	46.7%	20.0%	20.0%	26.7%
5	Excellent quality and excellent flow, very useful to patients	20.0%	46.7%	20.0%	20.0%

GQS, Global Quality Score. The global score discription of GQS was referred from Bernard et al.^14^

## Data Availability

The datasets generated and/or analyzed during the current study are available from the corresponding author upon reasonable request.
